# Mapping the reach of a rural Transitions Nurse Program for veterans with geographic information systems

**DOI:** 10.1186/s43058-020-00026-4

**Published:** 2020-03-19

**Authors:** Brigid Connelly, Lexus L. Ujano-De Motta, Chelsea Leonard, Ashlea Mayberry, Lynette Kelley, David Gaskin, Heather M. Gilmartin

**Affiliations:** 1 Denver/Seattle Center of Innovation for Veteran-Centered and Value Driven Care, VA Eastern Colorado Healthcare System, 1700 N. Wheeling St, Aurora, CO 80045 USA; 2 Health Management, Systems, and Policy, University of Colorado, School of Public Health, Aurora, CO 80045 USA

**Keywords:** Rural, Veterans, Implementation science, Geographic information systems

## Abstract

**Purpose:**

Rural Veterans who receive inpatient care at a Veterans Health Administration (VA) tertiary facility can face significant barriers to a safe transition home. The VA rural Transitions Nurse Program (TNP) is a national, intensive care coordination quality improvement program for rural Veterans. To communicate the reach of TNP into rural communities, we developed geographic information system (GIS) maps. This study evaluated TNP transitions nurse and site champion perceptions of GIS as a communication tool for illustrating the reach of TNP into rural communities.

**Methods:**

Using residence information for TNP enrollees, we built GIS maps using ArcGIS Enterprise, a mapping and analytics platform. Residential addresses were matched to Rural-Urban Commuting Area geographical categories. Transitions nurse and site champion perceptions of the local and national GIS maps were assessed through surveys and interviews. The data were analyzed using descriptive and content analytic methods to identify themes.

**Results:**

Transitions nurses and site champions perceived GIS maps as a valuable, easy to understand, acceptable, and appropriate communication tool to illustrate the reach of TNP into rural communities. Interviews revealed three common themes: a picture is worth a thousand words, the GIS maps are an effective communication tool, and the GIS maps revealed surprising and promising information.

**Conclusions:**

GIS is a useful communication tool to support to illustrate the reach of an intervention. The GIS maps engaged transitions nurses and site champions in discussion. The availability of open access software programs and publicly available location data will increase access to GIS for researchers and practitioners.

Contributions to the literature

- Communicating the reach of the Transitions Nurse Program (TNP) into rural communities is important for ongoing support and sustainment of program funding but it can be a challenge for programs to accurately calculate reach.
- This study evaluated stakeholder perceptions of geographic information systems (GIS) as a communication tool for illustrating the reach of TNP into rural communities**.**
- The study findings contribute to the field by confirming the utility of GIS as a healthcare communication tool, particularly when a barrier to care is geographic location.

## Background

Nearly 2.7 million Veterans live in rural communities and use the Veterans Health Administration (VA) for healthcare [1]. Access to care for this population is a challenge due to significant geographic distances between VA tertiary hospitals, located in urban settings, and VA clinics and hospitals, commonly located in rural settings [1, 2]. The transition for rural Veterans from hub VA hospitals back home to VA primary care clinics is complicated by communication and logistical barriers as well as limited health resources in rural communities [2]. Compared to the urban Veteran population, rural Veterans experience a higher disease prevalence and lower physical and mental quality-of-life scores [3]. The VA rural Transitions Nurse Program (TNP), a nurse-led intensive care coordination quality improvement project, was designed to address the geographical barriers to care rural Veterans experience after discharge from urban VA hospitals. A pilot run from 2014 to 2016 in Denver demonstrated increased rates of follow-up with rural primary care providers, and a trend towards fewer unplanned readmissions for Veterans enrolled in TNP [2]. Due to this, TNP was selected for national expansion to 11 VA hospitals by the VA Office of Rural Health [2, 4]. At each VA hospital, TNP is led by a transitions nurse, who completes the TNP intervention, and a site champion, who serves as a liaison between the transitions nurse and VA site leadership.

The spread and scale up of TNP across the VA was guided by the Reach, Effectiveness, Adoption, Implementation, and Maintenance (RE-AIM) framework [5]. The goal of RE-AIM is to encourage implementors and evaluators to pay attention to essential program elements that can improve the adoption and sustainment of effective, generalizable, evidence-based interventions. Understanding the degree to which TNP reaches those most at risk for poor transitions home was of great interest to VA leadership that supported the program locally. Reach, as defined by RE-AIM, refers to the proportion and risk characteristics of eligible persons who receive or are affected by a program [5]. Understanding the reach of TNP requires information on TNP Veterans as well as Veterans eligible for TNP, but not enrolled. However, detailed information on Veterans potentially eligible for TNP, but not enrolled, was challenging to collect due to the operational focus of the program. Sites were permitted to enroll high-risk Veterans that did not meet program criteria, if it was in the best interest of that Veteran. Therefore, any calculation of a denominator of reach was challenging and would not produce a reliable value. A TNP site champion suggested we investigate alternative methods to present the reach of TNP into rural communities.

One innovative tool to communicate such information is geographic information systems (GIS), a framework for gathering and analyzing complex information to display as maps. Within healthcare, GIS have efficiently identified disease patterns, examined relationships between factors such as cost, distance, and access to care, and integrated large amounts of data for planning and research questions [6]. GIS have guided policy, resource allocation, and continuation of research. In the context of public health, GIS maps have been valued for their presentation and communication properties. GIS maps convert complex, raw data into usable images that are accessible to a mixed and non-technical audience [7]. GIS have been used to assist in decision-making when location is a key factor of that decision [8]. Within the VA, GIS have helped hospital leadership and staff understand the distribution of Veteran patients, visualize practice patterns, and illustrate the potential barriers Veterans may face. This helped policy makers and healthcare staff to prioritize resource allocation and potentially save Veteran lives [9, 10]. This study aimed to evaluate transitions nurse and site champion perceptions of GIS as a communication tool for illustrating the reach of TNP into rural communities.

## Methods

### Design and population

We conducted a mixed methods evaluation of transitions nurse and site champion perceptions of the TNP GIS maps. The 11 transitions nurses that provide care coordination to rural Veterans and the 10 site champions that support implementation of TNP at each site were included. The principal investigator of TNP (HG) was a site champion at one site and the interviewer for this project. Transitions nurses and site champions were invited to participate in an online presentation of the 2018 TNP Outcomes Report and subsequent interviews via email calendar invite. The presentation and interviews were conducted over Skype video conferencing by the TNP principal investigator using a semi-structured interview guide format. The presentations included the reasons and interests in trialing GIS as a communication tool and potential uses of the maps. Transitions nurses and site champions were oriented to the maps, and time was given for a question and answer session prior to the interviews. The interviewer is PhD trained in mixed methods and had worked directly with the transitions nurses and site champions since 2016. The interviewer, transitions nurses, and site champions were the only ones on the call. The design and reporting of the qualitative data from this evaluation were performed per the Consolidated Criteria for Reporting Qualitative Research (COREQ) checklist [11]. In addition to the qualitative data, the Acceptability of Intervention Measures and Appropriateness of Intervention Measures [12] survey was administered and analyzed using descriptive statistics.

### Description of interview and survey

A semi-structured interview guide (Additional file 1: Appendix 1) was created by a PhD-trained qualitative researcher (CL) and pilot tested with the Clinical Director of Training for TNP (LK). The questions were designed to elicit transitions nurse and site champion perceptions of the GIS maps, understand how sites would use the maps, and to solicit suggestions to improve the visual presentation of the GIS maps. Each interview lasted 30 min and no follow-up interviews were conducted. Data saturation was discussed after each interview between the interviewer and the lead analyst (CL). No field notes were taken. The interviews were recorded using Skype video call and were transcribed verbatim. The transcripts were not returned to nurses or champions for comment or correction. Immediately after each presentation and interview, the Acceptability of Intervention Measures and Appropriateness of Intervention Measures [12] survey was emailed to each participant (Additional file 1: Appendix 2). The eight-item survey is a validated measure that assesses the acceptability and feasibility of implementation strategies such as GIS maps. The survey was administered using VA REDCap.

### Analysis

Transcripts were analyzed using deductive content analysis [13] in Microsoft Excel 16.3. A structured categorization matrix were developed to code the data based on the interview questions. All the interview responses were reviewed for content and correspondence to the following categories: perception of map, plans for using map, and suggestions for map. Only contents that fit the matrix of analysis were chosen from the data. The categories were discussed between the lead analyst and interviewer. Face validity of the categorized results was established by the Clinical Director of Training for TNP. Quotes were used to enhance the credibility of the research findings and demonstrate consistency between the data presented and the categories.

### Creation of TNP GIS maps

Creation of the GIS maps required ArcGIS mapping software available through the VA Office of Rural Health GeoSpatial Outcomes Division. The data were accessed and analyzed within the VA Informatics and Computing Infrastructure (VINCI) [14] development workspace using Microsoft SQL (Structured Query Language) Server, ArcGIS 10.5.x, and File Transfer Protocol. Within the VINCI workspace, a SQL code was written to pull information for Veterans enrolled in TNP between April 2017 and December 2018. This information included TNP Veterans’ addresses from the VA Corporate Data Warehouse (CDW) which were imported into ArcGIS as tabular data. The residential addresses were geocoded into latitude and longitude coordinates in order to show their relative spatial locations represented as data points on a map. Data were harmonized with Rural-Urban Commuting Area (RUCA) codes, using state and county Federal Information Processing Standard (FIPS) codes as a reference table to create a rurality layer. A map layer is a GIS database containing groups of point, line, or area (polygon) features representing a particular class or type of real-world entities such as customers, streets, or postal codes. The rurality layer was one of many geographical layers stacked to create a comprehensive map. Additional layers of contextual data such as state, county, census tract boundaries, major roadways, and interstates were downloaded as shapefiles from the US Census Bureau 2018 Topologically Integrated Geographic Encoding and Referencing (TIGER) geodatabase [15].

A national TNP map and 11 individual site maps were created as one-page reports that visually presented catchment areas, using color to represent the RUCA-based layers of urban, rural, and highly rural. TNP enrollees with missing addresses in CDW were excluded from the study.

## Results

**Fig. 1:**
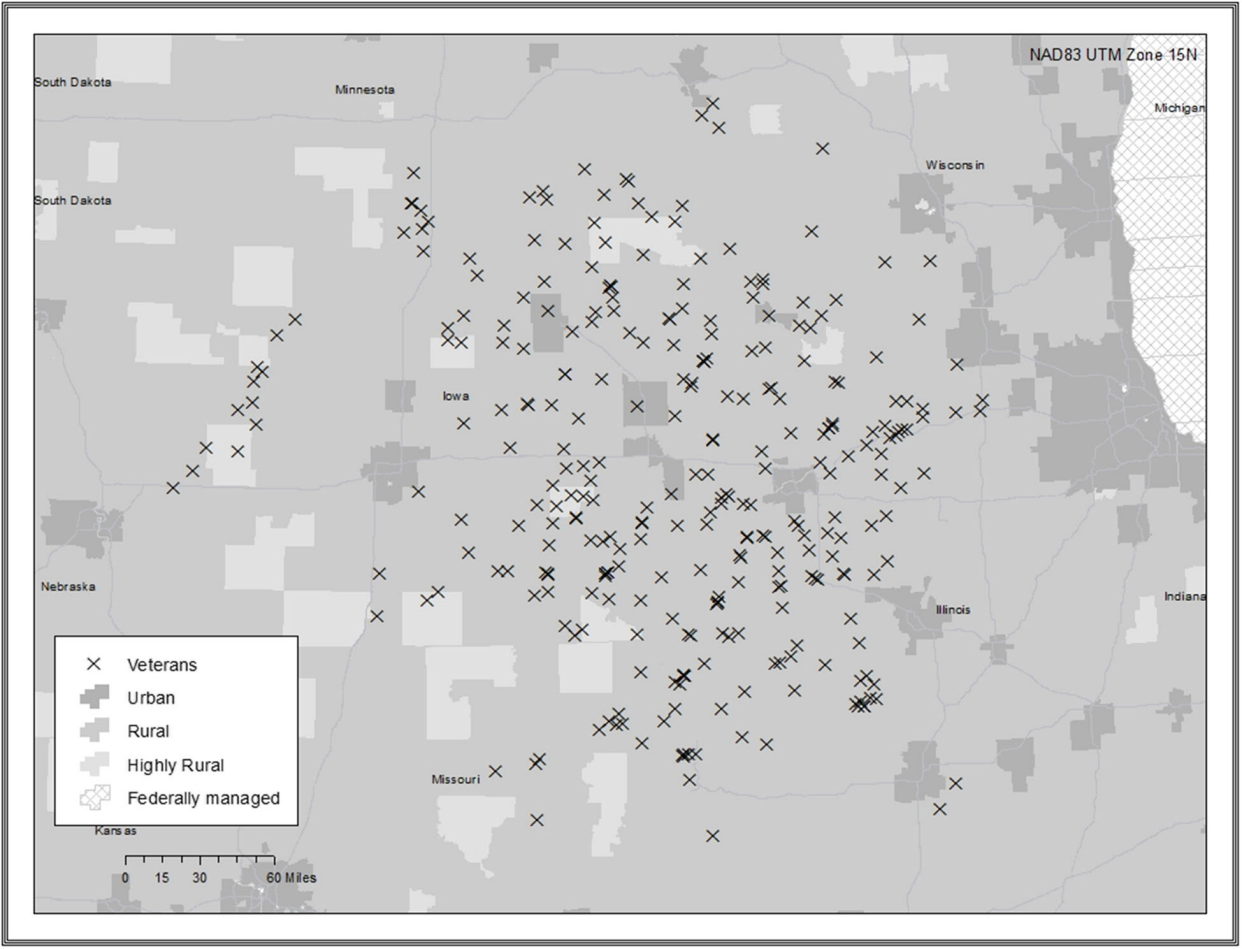
Large geographical spread

**Fig. 2:**
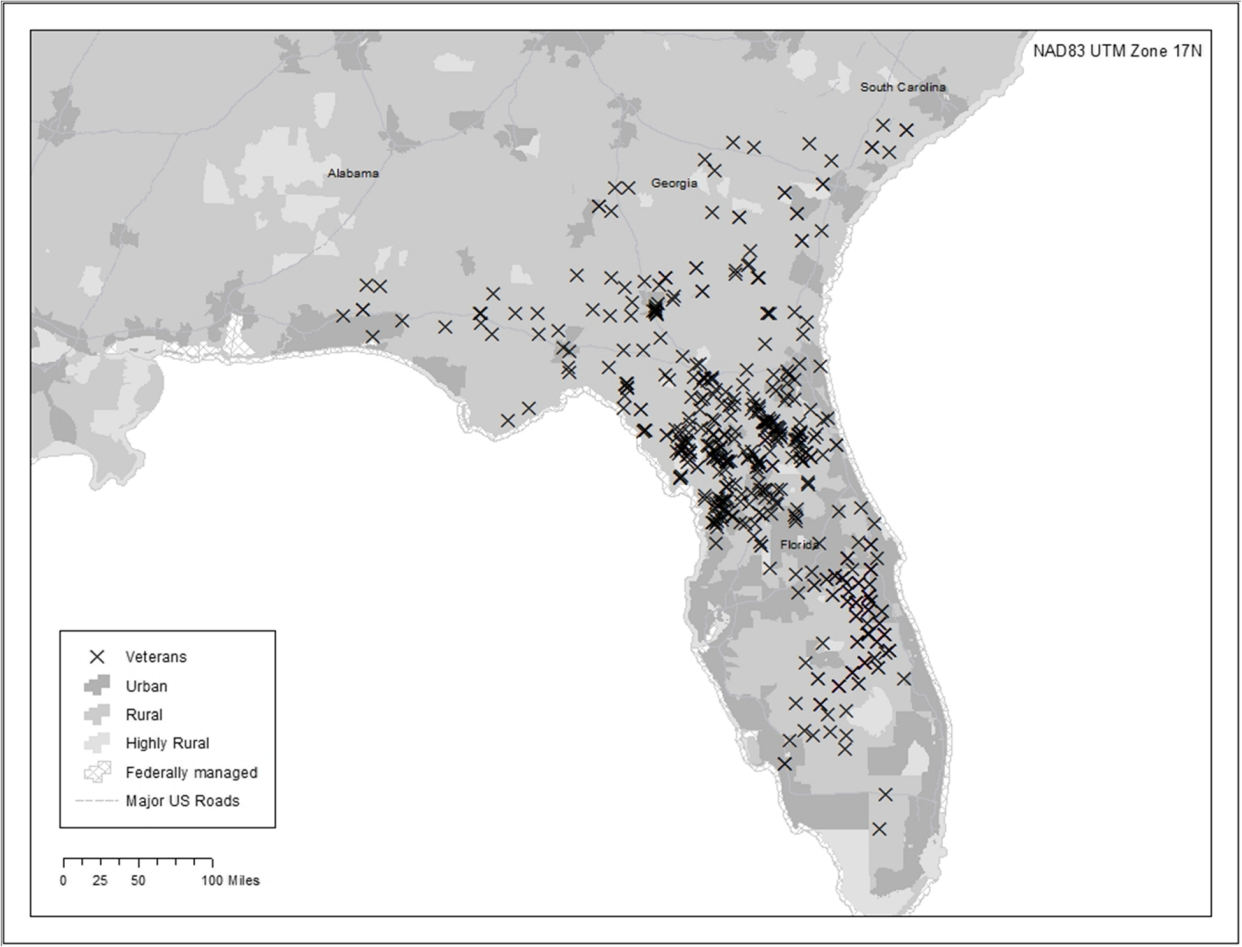
Concentrated geographical clumps. These are fictitious point maps that show the variation in distribution of patients at different sites. We have not used location data from patients enrolled in TNP in order to protect patient privacy and compliance with the Health Insurance Portability and Accountability Act. This map illustrates the level of detail we are able to share with sites

The TNP GIS maps communicated the reach of TNP across both rural and urban areas. Some maps communicated an even geographical spread of Veteran enrollees (Fig. 1), while others showed concentrated clusters of TNP Veterans (Fig. 2). The TNP population included in the study was 2499 Veterans, with 7 Veterans excluded due to missing addresses in CDW. These Veterans were excluded largely due to issues in the VA’s Planning Systems Support Group (PSSG), including a low-quality geocode “score” and a lag in the publication of the PSSG Geocoded Enrollee Files.

The 11 presentations and interviews were conducted with 11 nurses (10 females, 1 male), 9 physicians (3 females, 6 males), and 1 administrator (male). No one refused to participate; however, one interview (one nurse, one physician) was not recorded due to equipment malfunction, so data were not captured. Content analysis revealed three main themes related to transitions nurse and site champion perceptions and planned use of the GIS maps: (1) a picture is worth a thousand words, (2) GIS maps are a good communication tool, and (3) the GIS maps revealed surprising and useful information.

### A picture is worth a thousand words

Most transitions nurses and site champions indicated that the maps illustrated that TNP affects a large geographic area and is far reaching into rural communities. They indicated the maps were effective in communicating the reach of TNP and showed how TNP enrolls Veterans from a VA hospital’s entire catchment area. One participant indicated that the map illustrated the reach of TNP into underserved geographic regions, and another said that maps illustrated how far some Veterans lived from their VA hospital. One participant stated, “I think a picture is worth a thousand words. It shows that we have patients from our entire catchment area, you know, many of them are, you know, hours and hours away.” Another echoed this sentiment, and stated, “I love the map […] it definitely is eye-catching. It shows what kind of reach we have as a facility. I mean, look at those X’s all the way down in [rural location].” Yet another participant stated, “it’s fun to look at the map and see the severe isolation.”

### The GIS maps are a good communication tool

Transitions nurses and site champions felt the maps would be useful for communicating TNP to leadership as they are a nice way to visualize the far-reaching impact of TNP. They viewed the maps as a simple, yet visually pleasing method to visualize the reach of TNP and the extreme isolation of some rural Veterans. One said, “I think it’s a good advertisement for the program, to emphasize what our goal is.” Several sites said that they would use the map in presentations to local leadership. One said that the maps would help leadership “visualize the impact [of TNP] because […] we have a patient who may need this and this and this, but they’re in the middle of […] it will help for [leadership] to be aware of those distances and maybe how I [the Transitions Nurse] can help.”

### The GIS maps revealed surprising and useful information

Two sites were surprised by the data presented on the maps, which resulted in discussion and strategizing among the transitions nurse, site champion, and TNP principal investigator. One site was unaware of the high concentration of urban Veterans enrolled in their program. They stated, “I was surprised at that concentrated area there of our urban group. We have a lot of urban [patients].” During the call they discussed how to adapt their enrollment efforts to capture more rural Veterans. Another site was surprised they were enrolling Veterans outside of their facility catchment area. Though this was not a concern, the map gave them evidence that their TNP program benefited other VA hospitals. All of the transitions nurses and site champions stated the maps presented data they could use to help promote and advocate for TNP to leadership to support continuation of the program.

### Acceptability of Intervention Measures and Appropriateness of Intervention Measures

The Acceptability of Intervention Measures and Appropriateness of Intervention Measures [12] survey was completed by 17 of 18 transitions nurses and site champions. Respondents indicated very high acceptability (median = 4; 1–5 Likert scale) and appropriateness (median = 5; 1–5 Likert scale) of the GIS maps as a communication tool illustrating the reach of TNP (Table 1). Four respondents provided additional feedback in an open text box at the end of the survey. Two comments provided positive feedback (“Love it,” and “Good looking and helpful”), and two provided suggestions (“Add on key locations, like the community based outpatient clinics,” and “recommend putting the [VA hospital] on the map for the user to see relative distance from Veterans to the [VA hospital]—could also include community based outpatient clinic sites...”).

**Table 1:** Acceptability of intervention measures and appropriateness of intervention measures^10^ survey

Item (*n* = 17)	Median
The GIS meets my approval	4
The GIS map is appealing to me	4
I like the GIS map	4
I welcome the GIS map	5
The GIS map seems fitting	4
The GIS map seems suitable	5
The GIS seems applicable	5
The GIS map seems like a good match	5

*GIS* geographic information systems, *Survey Scale* 1–5 ascending Likert scale

## Discussion

This study aimed to evaluate transitions nurse and site champion perceptions of GIS as a communication tool for illustrating the reach of TNP into rural communities. Transitions nurses and site champions overwhelmingly rated the GIS maps as an acceptable, appropriate, and engaging communication tool. They found the information conveyed on the maps helpful to understand if TNP was benefitting the targeted, rural population. They indicated that the maps were easy to read and would be a useful communication tool to help leadership understand the reach of TNP into rural and highly rural communities.

The positive responses confirm the utility of GIS as a healthcare communication tool, particularly when a barrier to care is geographic location. These findings align with previous studies. Bazemore et al [16] used GIS maps in a primary care setting to integrate and analyze clinical and population data for vulnerable urban communities, showing service area and penetration of primary care clinics into these communities. These maps received overwhelmingly positive feedback from hospital leadership, who possess little to no previous GIS experience. The GIS maps were reported to keep sites engaged and enthused due to enhanced community comprehension, new ideas about data use for strategic planning and population management, and an array of applications to improve their clinical revenue. Given the positive response in our study and others, and the need for guidance to provide resources to rural communities [9], GIS maps should be considered as a best practice healthcare communication tool.

This work must be considered in the context in which it was conducted. The TNP GIS maps were created due to challenges in assessing the proportion and representativeness of individuals enrolled in TNP compared to Veterans potentially eligible for TNP, but not enrolled, as reach is traditionally calculated [3]. Though a noted limitation of this study, it is a common issue for implementation projects that permit sites to adapt program components, such as Veteran enrollment criteria, to real-world demands. Transitions nurses and site champions did not report that program success was impacted by the absence of traditional reach data. Further, the exclusion of Veterans due to missing addresses is a known challenge in GIS. However, our intent was to show the broad reach of TNP. Due to this, the exclusion of a small percentage of Veterans was not deemed a major limitation. Last, the requirement for individual patient location data, GIS technical skills, and analytic software to create the maps may limit the applicability of this approach in systems without this level of data or expertise. Fortunately, the healthcare systems in the USA are creating data warehouses to support this type of work. Plus, GIS training courses and software are readily available to healthcare researchers and operational leadership.

## Conclusion

GIS maps are a valuable communication tool in healthcare. GIS maps engaged transitions nurses and site champions in discussion regarding the reach of an intervention into rural communities. The maps were viewed as a useful tool, for they provided an alternative to tables or text explanations and revealed surprising and key information that guided program adaptations and sustainment efforts. The availability of location data stored in VA data warehouses and open access data analytic software programs makes GIS a feasible technology for researchers and practitioners who work with rural communities.

## Supplementary information

**Additional file 1.** Guiding Questions for 2018 TNP Outcomes Report Presentation with TN’s and Champions.

**Additional file 2.** Acceptability and Appropriateness of Intervention Measure (AIM) Survey Questions.

## Data Availability

The datasets used and/or analyzed during the current study are available from the corresponding author on reasonable request.

## Abbreviations

CDW: Corporate data warehouse
COREQ: Consolidated Criteria for Reporting Qualitative Research
FIPS: Federal Information Processing Standard
GIS: Geographic information systems
PSSG: Planning Systems Support Group
RE-AIM: Reach, Effectiveness, Adoption, Implementation, and Maintenance
RUCA: Rural-Urban Commuting Area
SQL: Structured Query Language
TIGER: Topologically Integrated Geographic Encoding and Referencing
TNP: Rural Transitions Nurse Program
VA: Veterans Health Administration
VINCI: VA Informatics and Computing Infrastructure
