# Supplementary material for: Mapping the reach of a rural Transitions Nurse Program for veterans with geographic information systems
Source: Implement Sci Commun. 2020 Mar 19;1:36. doi: 10.1186/s43058-020-00026-4 (PMC7427864; doi:10.1186/s43058-020-00026-4)
Supplement: Supplementary file 1 — Additional file 1. Guiding Questions for 2018 TNP Outcomes Report Presentation with TN’s and Champions. [file 43058_2020_26_MOESM1_ESM.docx]

Appendix 1.

**Guiding Questions for 2018 TNP Outcomes Report Presentation with TN’s and Champions**

The goal of these questions is to learn what the TN’s and Champions think about the Outcome Reports. We are interested in how well they understand the data, what they think the data means for the future of TNP, how they plan to use the information, if the report effectively presented the data, and what they think about the GIS maps.

We will ask these questions at the end of a phone call discussing the report. We will schedule 40-60 minutes for these phone calls and reserve the final 20 minutes for these questions.

***Grounded prompts: If responses are limited or require clarification, probes may be used to illicit more detailed responses. Probes should use words or phrases presented by the participant using one of the following formats:***

***1. What do you mean by ____________?***

***2. Tell me more about ____________.***

***3. Give me an example of ____________.***

***4. Tell me about a time when ____________.***

***5. Who __________?***

***6. When __________?***

1. What is your main take-away from this report?

2. How will you use the information in this report?

3. Please tell me about the GIS map.

**Important to probe here, especially if they provide a short response (e.g. It is a map that shows where patients live).

[If Needed/not mentioned in #2] What will you do with this map?

1. 4. Tell me about the format of this report.

[If Needed] Was the information easy to read and understand?
