# Supplementary material for: Mapping the reach of a rural Transitions Nurse Program for veterans with geographic information systems
Source: Implement Sci Commun. 2020 Mar 19;1:36. doi: 10.1186/s43058-020-00026-4 (PMC7427864; doi:10.1186/s43058-020-00026-4)
Supplement: Supplementary file 2 — Additional file 2. Acceptability and Appropriateness of Intervention Measure (AIM) Survey Questions. [file 43058_2020_26_MOESM2_ESM.docx]

Appendix 2

**Survey Questions**

**Acceptability of Intervention Measure (AIM)**

|  | Completely disagree | Disagree | Neither agree nor disagree | Agree | Completely agree |
| --- | --- | --- | --- | --- | --- |
| 1. The GIS map meets my approval. | ➀ | ➁ | ➂ | ➃ | ➄ |
| 2. The GIS map is appealing to me. | ➀ | ➁ | ➂ | ➃ | ➄ |
| 3. I like the GIS map. | ➀ | ➁ | ➂ | ➃ | ➄ |
| 4. I welcome the GIS map in the TNP program | ➀ | ➁ | ➂ | ➃ | ➄ |

**Intervention Appropriateness Measure (IAM)**

|  | Completely disagree | Disagree | Neither agree nor disagree | Agree | Completely agree |
| --- | --- | --- | --- | --- | --- |
| 5. The GIS map seems fitting to TNP | ➀ | ➁ | ➂ | ➃ | ➄ |
| 6. The GIS map seems suitable to TNP | ➀ | ➁ | ➂ | ➃ | ➄ |
| 7. The GIS map seems applicable to TNP | ➀ | ➁ | ➂ | ➃ | ➄ |
| 8. The GIS map seems like a good match to TNP | ➀ | ➁ | ➂ | ➃ | ➄ |

9. Is there anything else that you would like to tell us about the GIS map?

[open ended]
